# PD83 Defining The Role Of Expert Patients In Health Technology Assessment: Lessons From Uruguay’s National Workshop

**DOI:** 10.1017/S0266462325102109

**Published:** 2025-12-29

**Authors:** Ofelia Noceti, Andrea Gimenez

## Abstract

**Introduction:**

Patient involvement is essential for diversifying health technology assessment (HTA) and addressing global healthcare demands. Uruguay’s first national workshop engaged patients and caregivers to develop a consensus on the role and contributions of expert patients in HTA, fostering inclusivity and advancing decision-making processes.

**Methods:**

The workshop convened 25 representatives from 17 patient organizations, representing patients (72%) and caregivers (28%) from across Uruguay. Participants shared experiences through surveys and discussions, addressing key challenges, treatment impacts, and expectations. Consensus building activities defined expert patient roles, competencies, and training needs to ensure meaningful contributions to HTA.

**Results:**

The key challenges identified included disease acceptance, family and economic burdens, and treatment accessibility (72%). Participants emphasized the importance of user-friendly, accessible, and affordable health technologies. The essential roles of expert patients included contributing real-world testimonies (88%), creating patient-friendly summaries (76%), and participating in decision-making processes. While 76 percent had communication skills, 52 percent lacked critical analysis training, and 92 percent emphasized the need for education on HTA processes and technical report interpretation. Participants valued structured training and expressed strong motivation (83%) to improve patient representation in HTA.

**Conclusions:**

Uruguay’s workshop demonstrated the value of engaging patients and caregivers in HTA processes. The resulting consensus document highlighted the potential of expert patients to bridge gaps between technical assessments and real-world impact, promoting equity and inclusivity. These findings offer practical insights for strengthening patient involvement in HTA globally.

